# What is AI? Applications of artificial intelligence to dermatology†

**DOI:** 10.1111/bjd.18880

**Published:** 2020-03-29

**Authors:** X. Du‐Harpur, F.M. Watt, N.M. Luscombe, M.D. Lynch

**Affiliations:** ^1^ Centre for Stem Cells and Regenerative Medicine Faculty of Life Sciences and Medicine King's College London 28th Floor, Tower Wing, Guy's Hospital London SE1 9RT UK; ^2^ The Francis Crick Institute 1 Midland Road London UK; ^3^ St John's Institute of Dermatology Guy's Hospital London UK; ^4^ Okinawa Institute of Science and Technology Graduate University Okinawa 904‐0495 Japan

## Abstract

In the past, the skills required to make an accurate dermatological diagnosis have required exposure to thousands of patients over many years. However, in recent years, artificial intelligence (AI) has made enormous advances, particularly in the area of image classification. This has led computer scientists to apply these techniques to develop algorithms that are able to recognize skin lesions, particularly melanoma. Since 2017, there have been numerous studies assessing the accuracy of algorithms, with some reporting that the accuracy matches or surpasses that of a dermatologist. While the principles underlying these methods are relatively straightforward, it can be challenging for the practising dermatologist to make sense of a plethora of unfamiliar terms in this domain. Here we explain the concepts of AI, machine learning, neural networks and deep learning, and explore the principles of how these tasks are accomplished. We critically evaluate the studies that have assessed the efficacy of these methods and discuss limitations and potential ethical issues. The burden of skin cancer is growing within the Western world, with major implications for both population skin health and the provision of dermatology services. AI has the potential to assist in the diagnosis of skin lesions and may have particular value at the interface between primary and secondary care. The emerging technology represents an exciting opportunity for dermatologists, who are the individuals best informed to explore the utility of this powerful novel diagnostic tool, and facilitate its safe and ethical implementation within healthcare systems.

## Abstract 2

**What is already known about this topic?**

- There is considerable interest in the application of artificial intelligence to medicine.
- Several publications in recent years have described computer algorithms that can diagnose melanoma or skin lesions.
- Multiple groups have independently evaluated algorithms for the diagnosis of melanoma and skin lesions.

**What does this study add?**

- We combine an introduction to the field with a summary of studies comparing dermatologists against artificial intelligence algorithms with the aim of providing a comprehensive resource for clinicians.
- This review will equip clinicians with the relevant knowledge to critically appraise future studies, and also assess the clinical utility of this technology.
- A better informed and engaged cohort of clinicians will ensure that the technology is applied effectively and ethically.

Plain language summary available online

In the last decade, a combination of novel computational approaches, increases in available computing capacity and availability of training data has facilitated the application of powerful mathematical algorithms in the field of artificial intelligence (AI). This has led to dramatic advances in the performance of computers in tasks that have previously only been possible for humans. Methods that can make predictions of data without direct human intervention in the training process are referred to as machine learning. Image classification has been at the forefront of machine learning research, and as visual pattern recognition plays a larger role in dermatology than perhaps in any other medical specialties, early clinical applications of machine learning have been within this specialty.

## What are artificial intelligence and machine learning?

**Figure 1:**
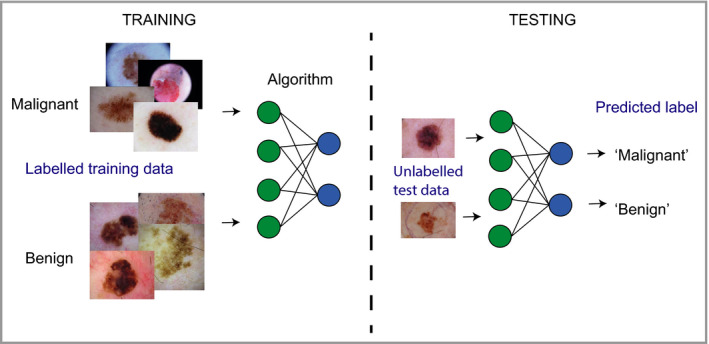
Schematic depicting how a machine learning algorithm trains on a large dataset to be able to match data to label (supervised learning), the performance of which can then be assessed.

AI is difficult to define precisely. In Alan Turing's seminal paper ‘Computing machinery and intelligence’, he proposed the well‐known Turing test, whereby a machine is deemed intelligent if it is indistinguishable from a human in conversation by an impartial observer.^1^ In modern parlance, artificial general intelligence refers to the ability of a machine to communicate, reason and operate independently in both familiar and novel scenarios in a similar manner to a human. This remains far beyond the scope of current methods and is not what is being referred to when the term ‘AI’ is commonly used. Most references to AI are now often used as an interchangeable term with ‘machine learning’ or ‘deep learning’, the latter being a specific form of machine learning that is discussed in more detail below (see Table 1 for a glossary of terms). Machine learning refers to algorithms and statistical models that learn from labelled training data, from which they are able to recognize and infer patterns (Figure 1).

**Table 1:** Essential terminology in the field of machine learning and artificial intelligence

Artificial intelligence (AI)	The ability of machines, such as computers, to simulate human intelligence
Machine learning	Algorithms and statistical models that are programmed to learn from data, therefore recognizing and inferring patterns within them. This enables computers to perform specific tasks without explicit instructions from a human operator
Supervised learning	Refers to machine learning tasks whereby the goal is to identify a function that best maps a set of inputs (e.g. image) to their correct output (label). This is based learning or training on prematched pairs. This is in contrast to unsupervised learning, where novel patterns such as groups or ‘clusters’ are identified in data without influence from prior knowledge or labelling
Overfitting	A common problem in machine learning where the model has high accuracy when tested on data from the same source as its training data, but its performance does not generalize to novel sources of data
Neural network	A form of supervised machine learning inspired by biology whereby data pass through a series of interconnected neurons, which are individually weighted to make predictions. During training, the data pass through the network in an iterative manner and the weightings are continually adjusted to optimize its ability to match label to data
Deep learning	Refers to a neural network with multiple layers of ‘neurons’ that have adjustable weights (mathematical functions)
Convolutional neural network	Refers to a type of neural network whereby the layers apply filters for specific features to areas within an image

**Figure 2:**
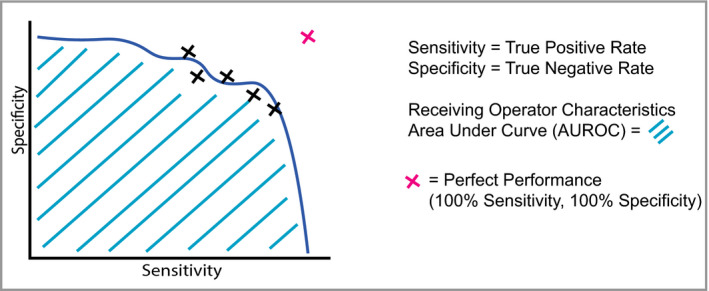
Schematic of a receiver operating characteristic (ROC) curve, which is a way of visualizing the performance of a trained model's sensitivity and specificity. Typically, machine learning studies will use ROC curves and calculations of the area under the curve (AUC or AUROC) to quantify accuracy. The dashed line represents the desired perfect performance, when sensitivity and specificity are both 100%; in this scenario, the AUC would be 1·0. In reality, there is a trade‐off between sensitivity and specificity, which gives rise to a curve.

Generally, during the training of a machine learning model a subset of the data is ‘held back’ and then subsequently used for testing the accuracy of the trained model. The accuracy of the model is assessed on this test dataset according to its accuracy in correctly matching an image to its label, for example melanoma or benign naevus. In any classification system there will be a trade‐off between sensitivity and specificity; for example, an AI system may output a probability score for melanoma between 0 and 1, and this would require the operator to set a threshold for the decision boundary. At a low threshold, a higher proportion of melanomas will be captured (high sensitivity) but there is a risk of classifying benign naevi as malignant (low specificity). As the threshold is increased, this would decrease the sensitivity, but increase the specificity (i.e. fewer benign naevi classified as melanoma). The behaviour of a machine learning classifier in response to changing the threshold can be visualized as a receiver operating characteristic (ROC) curve. The greater the area under the curve, the more accurate the classifier (Figure 2).

## Deep learning and neural networks

**Figure 3:**
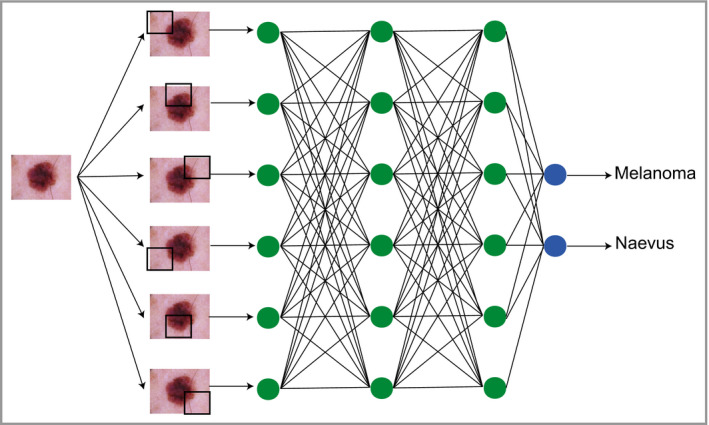
Schematic depicting how classification tasks are performed in convolutional neural networks. Pixel data from an image are passed through an architecture consisting of multiple layers of connecting nodes. In convolutional neural networks, these layers contain unique ‘convolutional layers’, which operate as filters. These filters work because it was recognized that the location of a feature within an image is often less important than whether that feature is present or absent – an example might be (theoretically) the presence or absence of blue‐grey veiling within a melanoma. A convolutional ‘filter’ learns a particular feature of the image irrespective of where it occurs within the image (represented by the black squares). The network is composed of a large number of hierarchical filters that learn increasingly high‐level representations of the image. These could in principle learn dermoscopic features similar to those described by clinicians, although in practice the precise features recognized are likely to differ from classic diagnostic criteria.

Neural networks (Figure 3) pass input data through a series of interconnected nodes (analogous to biological neurons). Each node functions as a mathematical operation (addition, multiplication, etc.), and a group of interconnected nodes within the network is referred to as a ‘layer’ within a network, with the overall structure of the layers being referred to as the ‘architecture’. During training, every node is adjusted and optimized through an iterative process called ‘backpropagation’,^2^, ^3^ allowing the neural network to improve its classification accuracy.

Neural networks with multiple ‘hidden layers’ of nodes (Figure 3) are referred to as ‘deep’ neural nets and perform ‘deep learning’. Although the concept of deep neural networks was described decades ago, lack of affordable and efficient computing power was a major limitation in being able to train them effectively. However, in 2013 it was recognized that graphical processing units (GPUs), originally designed for three‐dimensional graphics in computer games, could be repurposed to power the repetitive training required for neural networks.^4^, ^5^ Of note, convolutional neural networks (CNNs) are a specific form of deep learning architecture that have proven effective for the classification of image data. CNNs have massively increased in popularity as a method for computer‐based image classification after the victory of the GPU‐powered CNN AlexNet in 2012, which won the ImageNet competition with a top 5 error rate of 15·3%, which was a remarkable 10% improvement on the next best competitor.^5^

In the past few years, use of CNNs in classification tasks has exploded due to demonstrable and consistently superior efficacy and availability. Novel CNN architectures have been developed, improved and made available for public use by institutions with a high level of expertise and computational resources; examples of these include ‘Inception’ by Google and ‘ResNet’ by Microsoft. These architectures can be accessed using software such as TensorFlow (developed by Google) or PyTorch (developed by Facebook) and then trained further for a specific purpose or used in a novel application. A common approach would be to take a pretrained image recognition network architecture such as Inception, and specialize its application by inputting a specific type of image data. This process is referred to as transfer learning.

## The application of convolutional deep learning in dermatology

Classifying data using CNNs is now relatively accessible, computationally efficient and inexpensive, hence the explosion in so‐called ‘artificial intelligence’. In medicine to date, the main areas of application have been the visual diagnostic specialties of dermatology, radiology and pathology. Automating aspects of dermatology with computer‐aided image classification has been attempted in dermatology for over 30 years;^6^, ^7^, ^8^ however, previous efforts have achieved only limited accuracy. Although attempts have been made in recent years to use neural networks to diagnose or monitor inflammatory dermatoses,^9^, ^10^, ^11^ these have generally not been as successful or impressive as the networks constructed to diagnose skin lesions, particularly melanoma. Melanoma is therefore the focus of the remainder of this review, and Table S1 (see Supporting Information) summarizes these head‐to‐head comparison studies.^12^, ^13^, ^14^, ^15^, ^16^, ^17^, ^18^, ^19^, ^20^, ^21^

**Figure 4:**
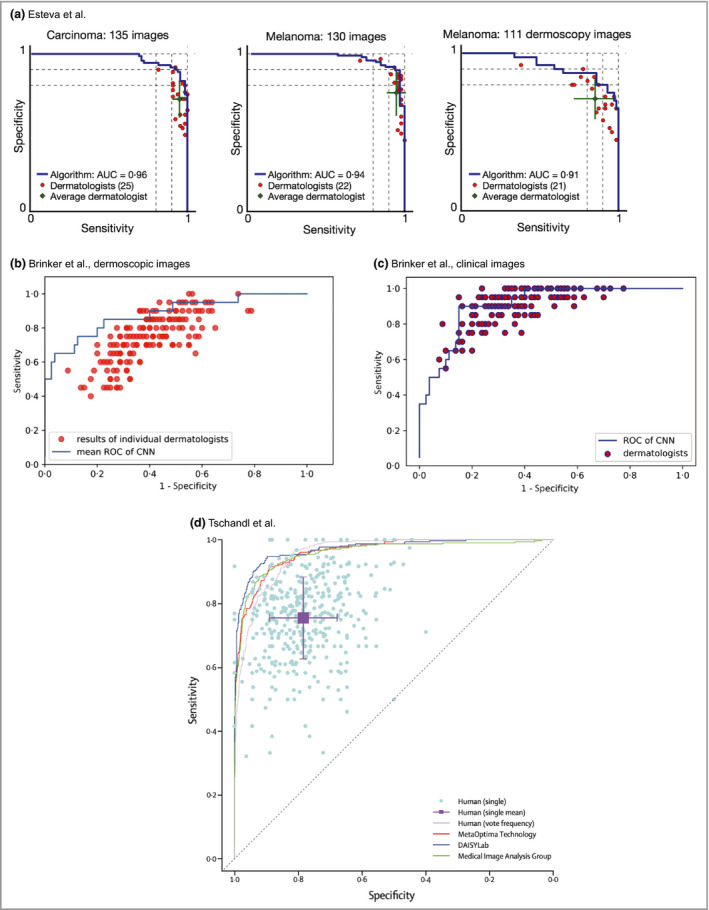
Receiver operating characteristic (ROC) curves from studies by Esteva *et al*.,^14^ Brinker *et al*.^19^, ^20^ and Tschandl *et al*.^21^ Most often, the dermatologists’ comparative ROC curves are plotted as individual data points. Lying below the curve means that their sensitivity and specificity, and therefore accuracy, are considered inferior to those of the model in the study. The studies all demonstrate that, on average, dermatologists sit below the ROC curve of the machine learning algorithm. It is noticeable that the performance of the clinicians in Brinker's studies (b, c), for example, is inferior to that of the clinicians in the Esteva study (a). Although there is a greater spread of clinical experience in the Brinker studies, the discrepancy could also be related to how the clinicians were tested. In both Brinker's and Tschandl's studies, some individual data points represent performance discrepancy that is significantly lower than data would suggest in the real world, which could suggest that the assessments may be biased against clinicians. AUC, area under the curve; CNN, convolutional neural network. All figures are reproduced with permission of the copyright holders.

In 2017, Esteva *et al*. published a landmark study in *Nature* that was notable for being the first to compare a neural network's performance against dermatologists.^14^ They used a pretrained GoogLeNet Inception v3 architecture and fine‐tuned the network (transfer learning) using a dataset of 127 463 clinical and dermoscopic images of skin lesions (subsequent studies have shown it is possible to train networks on significantly smaller datasets, numbering in the thousands). For testing, they selected a subset of clinical and dermoscopic images confirmed with biopsy and asked over 20 dermatologists for their treatment decisions. Dermatologists were presented with 265 clinical images and 111 dermoscopic images of ‘keratinocytic’ or ‘melanocytic’ nature, and asked whether they would: (i) advise biopsy or further treatment or (ii) reassure the patient. They inferred a ‘malignant’ or ‘benign’ diagnosis from these management decisions, and then plotted the dermatologists’ performance on the network's ROC curves with regards to classifying the keratinocytic or melanocytic lesions (which were subdivided as dermoscopic or clinical) as ‘benign’ or ‘malignant’ (Figure 4a). In both ‘keratinocytic’ and ‘melanocytic’ categories, the average dermatologist performed at a level below the CNN ROC curves, with only one individual dermatologist performing better than the CNN ROC curve in each category. This suggests that in the context of this study, the CNN has superior accuracy to dermatologists.

A recently published large study detailed in two papers by Brinker *et al*.^19^, ^20^ involved training a ‘ResNet’ model on the publicly available International Skin Imaging Collaboration (ISIC) database,^22^ which contains in excess of 20 000 labelled dermoscopic images and is required to meet some basic quality standards. This network was trained on over 12 000 images to perform two tasks: the first was to classify dermoscopic images of melanocytic lesions as benign or malignant (Figure 4b), and the second was to classify clinical images of melanocytic lesions as benign or malignant (Figure 4c). The dermatologists were assessed using 200 test images, with the decision requested mirroring that of the study of Esteva *et al*.: to biopsy/treat or to reassure. Additionally, the dermatologists’ demographic data, such as experience and training level, were requested.

The method used to quantify the relative performance also consisted of drawing a mean ROC curve by calculating the average predicted class probability for each test image (Figure 4b, c). The dermatologists’ performance for the same set of images was then plotted on the ROC curve. Barring a few individual exceptions, the dermatologists’ performance fell below the CNN ROC curves in both the clinical and dermoscopic image classifications. The authors also used a second approach, whereby they set the sensitivity of the CNN at the level of the attending dermatologists, and compared the mean specificity achieved at equivalent sensitivity. In the dermoscopic test, at a sensitivity of 74·1%, the dermatologists’ specificity was 60% whereas the CNN achieved a superior 86·5%.

As part of an international effort to produce technology for early melanoma diagnosis, in 2016 an annual challenge was established to test the performance of machine learning algorithms using the image database from the ISIC.^22^ A recent paper by Tschandl *et al*.^21^ summarizes the performance of the most recent competition in August to September 2018, and also compares the performance of the submitted algorithms against 511 human readers recruited from the World Dermoscopy Congress, who comprised a mixture of board‐certified dermatologists, dermatology residents and general practitioners (Figure 4d). Test batches of 30 images were generated to compare the groups, with a choice of seven diagnoses as multiple‐choice questions provided. When comparing all 139 algorithms against all dermatologists, dermatologists on average achieved 17 out of 30 on the image multiple‐choice questions, whereas the algorithms on average achieved 19. As expected, years of experience improved the probability for making a correct diagnosis. Regardless, the top three algorithms in the challenge outperformed even experts with > 10 years of experience, and the ROC curves of these top three algorithms sit well above the average performance of the human readers.

## Key biases, limitations and risks of automated skin lesion classification

Given that, remarkably, all of the published studies indicate superiority of machine learning algorithms over dermatologists, it is worth exploring the biases commonly found in these study designs. These can be categorized into biases that favour the networks and biases that disadvantage clinicians. With regards to the first category, it is first worth noting that in the studies described, the neural networks were generally trained and tested on the same dataset. This closed‐loop system of training and testing highlights a common limitation within machine learning called ‘generalizability’. On the occasions that generalizability has been tested, neural networks have often been found lacking. For example, Han *et al*. released their neural network, which was a Microsoft ResNet‐152 architecture trained on nearly 20 000 skin lesion images from a variety of sources as a web application.^15^ When Navarette‐Dechent *et al*. tested the network on data from the ISIC dataset, which the network had not previously been exposed to, its performance dropped from a reported area under the curve of 0·91, to achieving the correct diagnosis in only 29 out of 100 lesions, which would imply a far lower area under the curve.^23^ As algorithms are fundamentally a reflection of their training data, this means that if the input image dataset is biased in some way, this will have a direct impact on algorithmic performance, which will only be apparent when they are tested on completely separate datasets.

Another important limitation of the methodology used to compare AI models with dermatologists is that ROC curves, although a useful visual representation of sensitivity and specificity, do not address other important clinical risks. For example, in order to capture more melanomas (increased sensitivity), the algorithm may incorrectly misclassify more benign naevi as malignant (false‐positives). However, this could potentially lead to unnecessary biopsies for patients, which aside from patient harm would create additional demand on an already burdened healthcare system. There is evidence that dermatologists have improved ‘number need to biopsy’ metrics for melanoma in comparison with nondermatologists.^24^ The reporting of number need to biopsy would be a useful addition to studies such as that of Esteva *et al*.,^14^ as it would aid in the estimation of potential patient and health economic impact.

It is also worth noting that these datasets are retrospectively collated and repurposed for image classification training; this means that the images captured may not be representative in terms of the proportion of diagnoses, or in terms of having typical features. As neural networks are essentially a reflection of their labelled data input, this will undoubtedly have consequences on how they perform. However, given the lack of ‘real‐world’ studies, it is difficult to know how significant this is. When it comes to assessing clinicians using images from these datasets, this may also introduce an element of bias that disadvantages clinicians too, as lesions that were deemed worthy of capturing via photograph or being biopsied may not be representative of the lesion type. As a result, the sensitivity of clinicians diagnostically may be lower than in a normal clinic. This hypothesis for discrepancy in diagnostic accuracy was borne out in a recent Cochrane review, where the diagnostic sensitivity of dermatologists examining melanocytic lesions with dermoscopy was 92%,^25^ which is significantly higher than typically found in neural network studies. For example in Tschandl *et al*.'s web‐based study of 511 clinicians, the sensitivity of experts was 81·2%.^22^ The manner in which clinical decisions are inferred as ‘benign’ or ‘malignant’ also makes some assumptions that may not be accurate; for example, a dermatologist's decision to biopsy a lesion is a reflection of risk, not an outright ‘malignant’ classification.

From a safety perspective, there are two considerations that have yet to be addressed in the studies. Firstly, in order to ‘replace’ a dermatologist, an algorithm must be able to match the current gold standard for screening a patient's skin lesions. Currently, this is a clinical assessment by a dermatologist, who examines the lesion in the context of patient history and the rest of their skin. Published studies do not compare neural networks against this standard of assessment; they are only compared with dermatologists presented with dermoscopic or clinical images, sometimes with limited additional clinical information. Not only does this bias the studies against dermatologists, who are not trained or accustomed to make diagnoses without this information, it also represents a limiting factor in justifying their deployment in a clinical setting as a replacement for dermatologists. Fundamentally, it has not yet been demonstrated that they are equivalent to the standard of dermatological care currently provided to patients. A second important consideration is the fact that training data lack sufficient quantities of certain types of lesions, particularly the rarer presentations of malignancy, such as amelanotic melanoma.^15^ It is not yet clear how algorithms will perform when presented with entirely novel, potentially malignant lesions; this has rare but significant safety implications for patients.

From a legal perspective, an issue that has yet to be fully addressed is the lack of explainability by neural networks. Currently, it is not possible to know what contributes to their decision‐making process. This has led to criticisms and concerns that neural networks function as ‘black boxes’ with potential unanticipated and hard‐to‐explain failure modes. The European Union's General Data Protection Requirement specifies explainability as a requirement for algorithmic decision making, which is currently not achievable.^26^, ^27^ Algorithmic decision making also has uncertain status in the USA, where the Food and Drug Administration have advised that until there exists a body of evidence from clinical trials, clinical decisions suggested by AI ought to be considered AI guided, not AI provided, and liability would still rest with the clinician.^28^

## The AI‐integrated health service of the future?

There are attempts to deploy ‘AI’ technologies within the healthcare space within two main scenarios: direct to consumer or public, and as a decision aid for clinicians. The direct‐to‐consumer model already exists in some fashion; there are smartphone apps such as SkinVision, which enable individuals to assess and track their skin lesions. However, currently such apps do not make accountable diagnoses and usually explicitly state in their terms and conditions that they do not provide a diagnostic service, and do not intend to replace or substitute visits to healthcare providers. At present, it is not yet clear what the benefits and risks of such a tool are in terms of how frequently it provides false reassurance, and how frequently it recommends referral when this is not needed. Although health data democratization has benefits from the perspective of patient autonomy, it may be that this does not translate to better health outcomes and might instead lead to unnecessary concern and investigations. Moreover, fundamentally, healthcare is currently structured in such a way that responsibility and liability are carried by the provider and not the patient, and as such these apps do not have a clear‐cut position in healthcare infrastructure.

**Figure 5:**
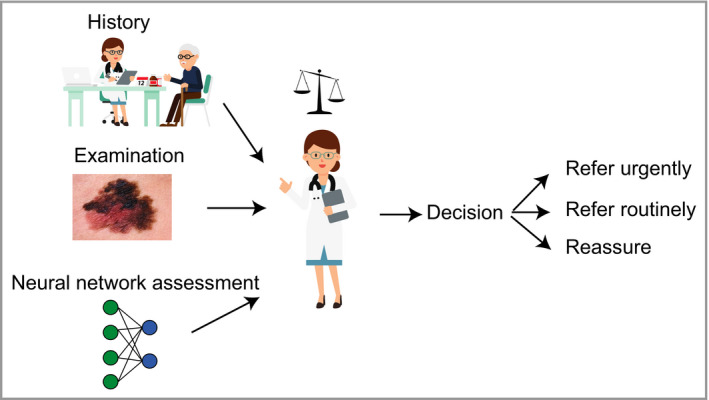
Schematic showing hypothetical use of a machine learning algorithm to help nonexpert clinicians risk‐stratify lesions to make clinical decisions. Clinicians routinely weigh up both the benefits and limitations of common diagnostic aids such as prostate‐specific antigen or D‐dimers. Currently, there are very few useful dermatological diagnostic decision aids available to nonexpert clinicians, as the diagnostic process is dominated by image recognition. Convolutional neural network could represent a new class of decision aid that could help nonexpert clinicians triage appropriately and narrow down their differential diagnosis.

The current social and legal framework of healthcare is better primed for incorporating AI as a decision aid for clinicians, particularly in enhancing decision making by nonspecialists (Figure 5). This could potentially be of great use in dermatology services due to the ever‐growing burden of skin cancer. In the UK, there is a long‐standing shortfall of consultant dermatologists, and current workforce planning is insufficient to address this. The volume of skin cancers has a knock‐on effect on patients with chronic inflammatory skin diseases, essentially reducing their access to dermatologists.

Dermatologists are also aware that generally, a high proportion of referrals to dermatology with suspected skin cancer on the urgent ‘2‐week wait’ pathway do not require further investigation and are actually immediately discharged. Many of the lesions falling into this category are easily recognized by dermatologists, but are not easily recognized by nonspecialists. One could hypothesize that CNN‐based applications can aid a general practitioner service in triaging skin lesions more effectively, and ensure that patients are managed by the appropriate clinical services. Having a clinical user also mitigates many of the risks and limitations inherent to CNN‐based technologies, improving both the safety profile and the patient experience.

The recently published Topol Review on ‘Preparing the healthcare workforce to deliver the digital future’ states that ‘to reap the benefits, the NHS must focus on building a digitally ready workforce that is fully engaged and has the skills and confidence to adopt and adapt new technologies in practice and in context’. It also concludes that ‘the adoption of technology should be used to give healthcare staff more time to care and interact directly with patients’.^29^ In the context of dermatology, this very much holds true. Technology adoption could improve clinical pathways, and enable our neediest patients to access dermatology services more efficiently. It is unlikely that they will threaten our profession; in reality they represent an opportunity for personal learning, service improvement and leadership that could be transformative for our future healthcare system.

## Supporting information


**Table S1** Comparative studies between artificial intelligence algorithms and dermatologists obtained from studies published up until June 2019.
