# Supplementary material for: What is AI? Applications of artificial intelligence to dermatology
Source: Br J Dermatol. 2020 Mar 29;183(3):423–30. doi: 10.1111/bjd.18880 (PMC7497072; doi:10.1111/bjd.18880)
Supplement: Supplementary file 1 — Table S1 Comparative studies between artificial intelligence algorithms and dermatologists obtained from studies published up until June 2019. [file BJD-183-423-s001.docx]

Summary Table of comparative studies between AI algorithms and dermatologists obtained from studies published up until June 2019

| **Author, year** | **Architecture** | **Proposed aim of model** | **Input data for transfer learning** | **Test data provided to CNN and dermatologists** | **Comparison method** | **Network performance, and comparison result:** |
| --- | --- | --- | --- | --- | --- | --- |
| Esteva et al, 2017[^1^](https://paperpile.com/c/a6S94I/MytT) | GoogLeNet Inception v3 | Classification of keratinocytic or melanocytic lesions into benign or malignant (clinical and dermoscopic images) | 127,463 training and validation images, with 2032 disease labels. | Clinical (265) and dermoscopic (111) images confirmed with biopsy | Assessed against 21 dermatologists who were asked to decide to ‘biopsy/treat the lesion or reassure the patient’, inferring from this decisions regarding benign / malignant. | **Network:**  Carcinoma: AUC ROC 0.96  Melanoma: AUC ROC 0.94  Dermatoscopic melanoma: AUC ROC 0.91  **Dermatologists:**  In the 3 categories, only 1 dermatologist out of the 21-25 dermatologists were above the ROC curve of CNN |
| Marchetti et al, 2018[^2^](https://paperpile.com/c/a6S94I/ODLw) | A variety of machine learning fusion algorithms utilising predictions from 16 algorithms submitted in 2016 ISIC challenge | Classification of lesions as benign or malignant | Not applicable | 100 randomly selected dermoscopic images (half of which were melanomas) from the test dataset provided (n=379) | Eight dermatologists were asked to classify the lesions in the 100 images as benign or malignant and this was assessed as primary outcome. They were additionally asked about their management decision. | **Top performing fusion network:**  Sensitivity: 70%  Specificity at 82% sensitivity on ROC curve 76%  ROC AUC: 0.86  **Dermatologists average:**  **Classification:**  Sensitivity 82%  Specificity 59%  ROC AUC: 0.71  **Management:**  Sensitivity 89%  Specifity 47%  ROC 0.68 |
| Haenssle et al, 2018[^3^](https://paperpile.com/c/a6S94I/77mL) | GoogLeNet Inception v3 | Classification of dermoscopic melanocytic lesions into benign or melignant | Number not provided, but dermoscopic images from co-operating dermatologists and the International Skin Imaging Collaboration (ISIC) database were utilised. Labels were by determined dermatologists, or biopsy-proven (proportion not stated). | A test set of 300 images comprising 20% melanoma and 80% benign lesions were obtained from the validated image library at Department of Dermatology, University of Heidelberg. Two experienced dermatologists prospectively selected 100 images of set-300 for an increased diagnostic difficulty for use in testing network vs dermatologist | Benign or malignant classification of 58 dermatologists who were categorised by experience in dermoscopy. Dermatologist were also asked what their management would be (excise, short-term follow-up, discharge)  2-level approach  Level 1: Dermoscopic data alone  Level 2: Dermoscopic data plus clinical information and close-up image (4 weeks after level 1) | **Network:**  Classification AUC ROC 0.86  **Dermatologist**  Level 1 classification AUC ROC: 0.79  Level 2 classification AUC ROC 0.82  It is worth noting that the ROC for management (which infers decision about benign/malignant) differs considerably to classification. Notably, the dermatologists’ average sensitivity is 98.6%, which is higher than the 95% sensitivity of the network, even when tested on larger numbers of data. |
| Han et al, 2018[^4^](https://paperpile.com/c/a6S94I/UzbJ) | Microsoft ResNet-152 model | Classify clinical images of 12 skin lesions | 19,398 proprietary images | 480 random images selected from ‘Asan’ dataset and Edinburgh dataset. | Individual AUC ROCs calculated for all 12 diagnoses. Network assessed by 3 different test datasets (1 Asian, 2 Caucasian), although its results from only ⅔ fully described in paper.  16 dermatologists provided with image and multiple choice question for the 12 diagnostic categories. | **Network:**  Asan AUC 0.91  Edinburgh AUC 0.89  **Dermatologists:**  Individual performance (ROC) plotted on network’s ROC curves for correctly identifying BCC, intraepithelial carcinoma, squamous cell carcinoma and melanoma within the Asan and Edinburgh datasets. Average value of 16 dermatologists’ performance found to be inferior or equivalent at best. |
| Rezvantalab et al, 2018  [^5^](https://paperpile.com/c/a6S94I/nVvy) | DenseNet 201, ResNet 152, Inception v3, InceptionResNet v2 | Classification of 8 diagnostic categories of skin lesions | 10015 dermoscopic images from ISIC dataset, 200 dermoscopic images from PH2 dataset. | Not described | Not described | **Network (best performing examples):**  Resnet 152: ROC AUC% for melanoma 94.4%  DesnseNet 201: ROC AUC% for BCC 99.3%  **Dermatologist:**  Melanoma ROC AUC% 82.26%  BCC ROC AUC% 88.92% |
| Fujisawa et al, 2018  [^6^](https://paperpile.com/c/a6S94I/fZjt) | GoogLeNet | 3-level classification:  Level 1: Benign or malignant skin lesion, Level 2: Benign / malignant and epithelial / melanocytic  Level 3: 14 classifications | 4867 clinical images obtained from 1842 patients from University of Tsukuba. 14 diagnoses, a mix of malignant and benign | Random selection of 140 images from the training data, each dermatologist received a different 140 images so in total 1820 images were classified by dermatologists and 1142 by the network. | 13 board certified dermatologists, 9 dermatology trainees were asked to perform 1st-level classification, then 3rd-level classification | **Results reported as true positive percentage (sensitivity)**  **Network:**  Level 1: 92.4%± 2.1%  Level 3: 74.5%± 4.6%  **Board-certified dermatologists:**  Level 1:85.3%± 3.7%  Level 3: 59.7%± 7.1%  **Trainees:**  Level 1: 74.4%±6.8%  Level 3: 41.7%±12.0% |
| Tschandl et al, 2019[^7^](https://paperpile.com/c/a6S94I/fBmH) | InceptionResNetV2, InceptionV3, Xception, ResNet50. Two CNN (one trained on dermoscopic, another on clinical images) were combined and assessed against humans | Classification of non-pigmented skin lesions as cancerous, and also by specific diagnosis | 7895 dermoscopic, 5829 close-up images taken by one clinican at a primary skin cancer clinic. | CNNs were validated against all 2072 test images; not tested like-for-like against dermatologists. | 95 human raters (including 62 board-certified dermatologists), divided into 3 groups according to dermoscopy experience. All rated 50 random cases from the 2072-image test set as benign/malignant, asked to make a specific diagnosis and suggest management. | **Network:**  cCNN AUC: 0.742  cCNN sensitivity at 51.3% specificity in detecting skin cancer: 80.5%  cCNN frequency of achieving correct specific diagnosis: 37.6%  **Humans:**  AUC: 0.695  Sensitivity and specificity: 77.6%, 51.3%  Frequency of achieving correct specific diagnosis: 33.5% |
| Brinker et al, 2019[^8,9^](https://paperpile.com/c/a6S94I/HxiE+smVE) | ResNet50 | 1. Classification of dermoscopic melanocytic lesions as benign or malignant  2. Classification of clinical melanocytic images as benign or malignant | 12378 dermoscopic images from ISIC database  Clinical images from MED-NODE database | 100 unseen test images subsetted from ISIC and MED-NODE. | 1. 157 dermatologists from 12 hospitals were shown 100 images from ISIC database and asked to identify melanoma vs naevi  2. 145 dermatologists shown 100 images from MED-NODE database. Management decision (treat vs reassure) was asked. | **1. Dermoscopic**  **Network:**  At a sensitivity of 74.%1, specificity of 86.5% was achieved  **Dermatologists:**  Mean sensitivity 74.1%  Mean specificity 60%  Average dermatologists’ performance falls below ROC curve of CNN across all levels of experience  **2. Clinical images**  **Network:**  At equivalent sensitivity of 89.4%. Specificity 68.2%  **Dermatologists**:  Mean sensitivity 89.4%, specificity 64.4%  ROC curve of CNN on par with ROC of dermatologists |
| Tschandl et al, 2019[^10^](https://paperpile.com/c/a6S94I/O6Yl) | Summary of performance of machine learning algorithms in ISIC 2018 melanoma challenge | 1. Classification of melanocytic lesions into 7 specific diagnoses | 10015 images from ISIC database (as part of 2018 challenge) | Batches of 30 randomly selected lesions from test set; these included images from sources that did not contribute to training set. | 511 readers (283 board-certified dermatologists, 118 residents, 83 general practitioners) | On average the algorithms achieved a mean of 2.01 more correct diagnoses out of 30 test images, compared to the human participants  **Networks:**  Mean sensitivity of top 3 algorithms: 86.2%  **Humans:**  Mean sensitivity of experts (>10 years experience): 81.2% |
